# Investigation of the Dislocation Density of NiCr Coatings Prepared Using PVD–LMM Technology

**DOI:** 10.3390/ma16227234

**Published:** 2023-11-20

**Authors:** Guoqing Song, Wentian Wei, Bincai Shuai, Botao Liu, Yong Chen

**Affiliations:** College of Mechanical Engineering, University of South China, Hengyang 421101, China; sgq9807@163.com (G.S.); shuaibc920@163.com (B.S.);

**Keywords:** physical vapor deposition, laser micro-melting, NiCr coating, molecular dynamics, dislocation density

## Abstract

Micron-sized coatings prepared using physical vapor deposition (PVD) technology can peel off in extreme environments because of their low adhesion. Laser micro-melting (LMM) technology can improve the properties of the fabricated integrated material due to its metallurgical combinations. However, the microstructural changes induced by the high-energy laser beam during the LMM process have not been investigated. In this study, we used the PVD–LMM technique to prepare NiCr coatings with a controlled thickness. The microstructural changes in the NiCr alloy coatings during melting and cooling crystallization were analyzed using molecular dynamics simulations. The simulation results demonstrated that the transition range of the atoms in the LMM process fluctuated synchronously with the temperature, and the hexagonal close-packed (HCP) structure increased. After the cooling crystallization, the perfect dislocations of the face-centered cubic (FCC) structure decreased significantly. The dislocation lines were mainly 1/6 <112> imperfect dislocations, and the dislocation density increased by 107.7%. The dislocations in the twinning region were affected by the twin boundaries and slip surfaces. They were plugged in their vicinity, resulting in a considerably higher dislocation density than in the other regions, and the material hardness increased significantly. This new technique may be important for the technological improvement of protective coatings on Zr alloy surfaces.

## 1. Introduction

Zr alloys are utilized in nuclear reactor fuel cladding due to their excellent low neutron absorption rate. However, in the event of a coolant accident, Zr alloy is prone to oxidation reactions with water vapor which can produce a large amount of H_2_ and cause an explosion [1,2]. Therefore, it is necessary to prepare a protective coating on its surface to improve its corrosion resistance. Meanwhile, NiCr coatings exhibit excellent protective properties and promising applications in accident tolerant fuels (ATFs) [3,4,5].

The metal coatings on Zr alloy surfaces are usually prepared using PVD technology, which produces a dense and uniform coating [6]. The coating thickness can be precisely controlled by controlling the deposition duration. Nevertheless, because the film–substrate bonding mode of the coating prepared using PVD technology is physical, the bonding strength is low, and the coating can easily blister and fall off in a high-dose radiation environment [6,7,8,9]. LMM technology remelts and diffuses the coating and a portion of the substrate and fuses them to each other after condensation, creating a metallurgical bond that significantly improves the adhesion of the film–base. However, its high energy input can easily deform the workpiece, and detrimental effects such as coating cracks and significant element diffusion may occur [10,11,12,13,14]. The dislocation pileups can lead to stress concentrations and lattice mismatches. These dislocations are generally plugged near the grain boundaries, which causes the dislocation density to increase, resulting in an increased hardness. When the dislocation plugging reaches a certain level, the stress concentration causes the dislocations to break through the grain boundaries. Plastic deformation or cracking can occur because of the massive accumulation of dislocations [15,16,17,18]. Molecular dynamics relies on Newtonian mechanics to simulate the motion of molecular systems, combining theoretical methods and computer technology to obtain the evolution of the microstructure during the formation of material. Therefore, it is used to study the materials properties at the atomic level. Molecular dynamics simulations may be used to analyze the evolution of the dislocations resulting from LMM treatment. Indeed, many studies addressing the evolution of the crystals and defects of materials in complex environments have been carried out based on molecular dynamics simulations, and the simulation results have been verified via experiments [19,20,21,22,23,24,25,26,27,28].

New PVD–LMM technology was applied to treat the surface of the Zr alloys, with the aim of obtaining a functional coating that could dramatically improve the corrosion resistance and mechanical properties of the surfaces of the Zr alloys. In our work, the NiCr alloy coatings were prepared on Zr alloy surfaces by combining the advantages of the PVD and LMM techniques. The changes in the microstructure of the coating during the LMM process were analyzed using molecular dynamics, and the mechanical and chemical properties of the coating were verified experimentally. After LMM treatment, the dislocation density in the coating increased dramatically, and the dislocation aggregated at the twin crystals. Meanwhile, the adhesion and corrosion resistance of the coating were improved. We conducted a preliminary verification of the method for preparing a high-adhesion and corrosion resistance coating, which has shown promising feasibility. The coatings prepared using this technique will be beneficial for improving the service life of Zr alloys in extreme environments.

## 2. Experiment

### 2.1. Materials and Methods

A Zr alloy sheet (Zr-1Nb) was used in the experiment. A standardized specimen with dimensions of 20 mm × 20 mm × 5 mm were cut using wire cut electrical discharge machining (WEDM). The purity of the NiCr alloy target (Beijing Hezong Technology Co., Ltd. Beijing, China) was 3N (Ni: Cr = 70: 30 at.%), and the target impurity content is shown in Table 1. The surface of the Zr alloy was sanded using 2000# sandpaper (Stabcke, Melle, Germany), ultrasonically cleaned with alcohol for 30 min, and subsequently rinsed with deionized water. Finally, the sample was placed in a drying oven at 50 °C for 20 min to prepare for coating. A TSU-650 multifunctional coating machine (Beijing Technol Co., Ltd. Beijing, China) was used. The specific deposition process is presented in Table 2, and the coating deposition thickness was 18 μm. An XL-F300 fiber laser (Guangzhou Xinglai Laser Technology Co., Guangzhou, China) was used in the experiment, and the laser processing parameters are listed in Table 3. The samples were heated at 300 °C before LMM treatment.

To test the hardness of the sample using the HVS-1000AV microhardness tester (Advantech. Co., Ltd., Shanghai, China), 10 points were randomly selected on the surface of the sample, and an average value of the results was obtained. The applied load was 200 g, and the loading time was 10 s. The cross-sections of the samples were cut using WEDM and subsequently polished for analysis. The morphology of the samples was analyzed using HitachiSU-8000 SEM (Hitachi, Tokyo, Japan), and the element distribution of the cross-section was analyzed using EDS. The samples were sliced using the focused ion beam (FIB) technique, and the microstructure of the LMM samples was analyzed using TEM (FEI Talos F200 × G2) at an accelerating voltage of 200 kV. The coating adhesion was tested using an automatic scratch tester (WS-2005). The corrosion resistance of the coatings was tested using the electrochemical corrosion workstation (CS2350H) with an electrolyte of 3.5 wt.% NaCl.

### 2.2. Simulation and Calculation

Molecular dynamics simulations solve for each particle in the system by establishing Newtonian kinetic equations, statistically obtains the coordinates and momentum of each particle at each moment in the system operation, and elucidates the motion characteristics of the atoms in the system. All the atoms in the system follow Newton’s second law, which is calculated as follows.

(1) $$F_{i}(t)=m_{i}a_{i}(t),$$

where i is the particle number, F_i_(t) is the external force on the particle, m_i_ is the weight of the particle, and a_i_(t) is the acceleration of the particle as it moves.

The embedded-atom method (EAM) potential has been shown to accurately characterize a large number of metal properties based on the electron density. This helped solve the issue of not being able to define the volume in the potential function because of the energy due to the subjective factor. The main idea of the model is to consider each atom within the system as a guest atom, and the sum of the interaction potentials and embedding energies among the atoms is the energy of the system [29,30]. The cohesion energy (E*_coh_*) formula is as follows.

(2) $$E_{coh}=\sum_{i}F_{i}\left(\rho_{i}\right)+\frac{1}{2}\sum_{\begin{array}{c} i,\;\;\;j\\ i\neq j \end{array}}\emptyset_{ij}(r_{ij}),$$

(3) $$\rho_{i}=\sum_{j\left(j\neq i\right)}f_{j}(r_{ij}),$$

where $F_{i}\;$ is the embedding energy of the atom, $\rho_{i}$ is the electron density of the atom, $\emptyset_{ij}$ is the short-range two-body potential function, and $r_{ij}$ is the distance between atom i and atom j. The total energy of the system was obtained by summing each atom. The EAM potential simulated the mechanical properties, stacked laminar dislocations, solid-phase changes, surface structures, and dislocation densities of some of the metals, and the interatomic interactions described were consistent with the experimental data. The potential function was the EAM potential of NiCr [31]. Gibbs proposed a systems theory, which refers to the collection of systems with consistent structures and properties, but with states of motion that are independent of each other in an environment with certain macroscopic conditions [32]. The NPT system can control the pressure and temperature changes in this system during molecular dynamics simulations, which is mainly used for phase transformation and the crystallization of solid structures. Therefore, in this study, the NPT system was used.

**Figure 1:**
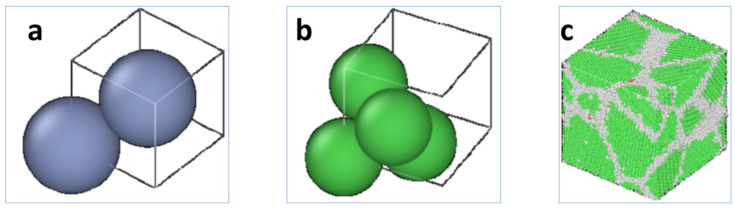
Modeling of the atomic crystal structure: (**a**) BCC structure of a Cr atom; (**b**) FCC structure of a Ni atom; (**c**) single-crystal poly cellular model of a NiCr alloy.

The structural phase transitions and crystallization processes of the coatings were investigated using isothermal isobaric system synthesis (NPT). The simulation calculations were carried out using the LAMMPS open-source software (12 Dec 18), and the results were visually analyzed using OVITO. VScode was used for programming the software, the LAMMPS text language, and the LAMMPS program self-test. Based on the lattice constants of the Ni and Cr atoms, Atomsk was used to establish an atomic crystal structure model (Figure 1). The single-cell structure of the Ni atoms was constructed based on the lattice constant of Ni (3.51). A 10 nm × 10 nm × 10 nm cell was built and filled with Ni atoms to form a supercell with an FCC structure. The replacement of 30% of the Ni atoms in the supercell with Cr atoms resulted in a body-centered cubic (BCC) structure totaling 92,679 atoms. In the NiCr alloy simulations, the FCC structure dominated. The remainder of the cluster types were referred to as “other” and were used to calibrate the initial velocity and initial position of the particles in the model system. The metal was chosen as the base unit in the simulation, and the atomic properties were used as the atomic unit. The energy minimization method was applied during relaxation to set the initial positions of the atoms and the initial kinetic energies so that the system atoms were in equilibrium. The analysis of the NiCr alloy melting point used the JMatPro software (JMatPro V11.0), while the system and temperature were controlled using a Gaussian temperature control method. The output step was 0.002 ps and the number of output steps was 10^6^. The cooling rate was three times the heating rate [32].

## 3. Results and Analysis

**Figure 2:**
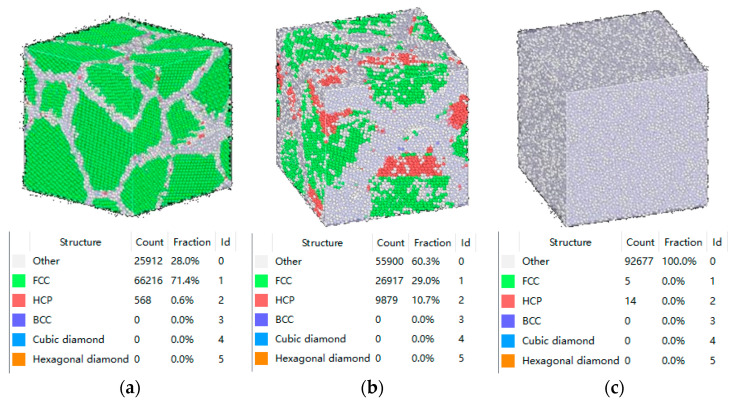
Heating and melting process of the NiCr alloy: (**a**) the warming process prior to the melting; (**b**) the melting process; (**c**) the fully melted state.

**Figure 3:**
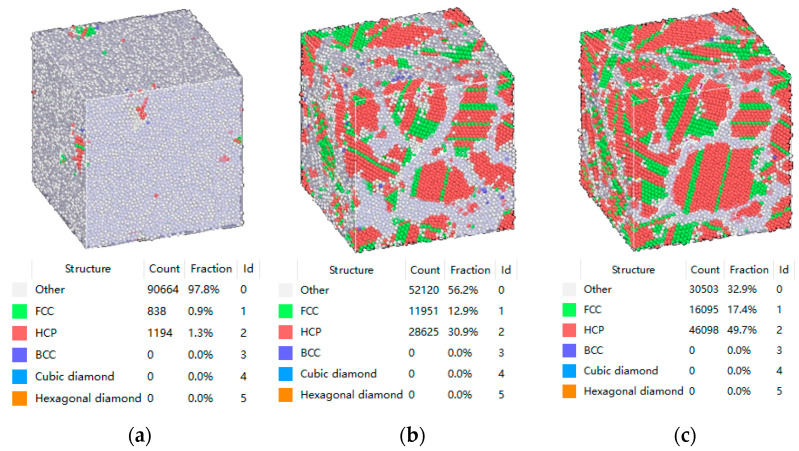
Cooling crystallization process of the NiCr alloy: (**a**) the fully melted state; (**b**) the solidification process; (**c**) the cooling process after solidification.

Figure 2 shows the melting process of the alloy system, and Figure 3 shows the cooling crystallization process. With increasing temperature, the internal energy of the atoms in the system continuously increased, and the amplitude of motion gradually became larger and free of particles. Meanwhile, the grain boundaries in the system widened, the FCC configuration was destroyed, and the grain boundaries were blurred. After reaching the melting point, the crystal structure underwent a phase change, and the atoms were in a disordered state. When the temperature decreased, the energy of the system decreased, and the atomic energy of the atoms within it also decreased. During solidification, the number of atoms in the FCC and HCP crystal structures increased, whereas those in the other crystal structures gradually decreased. The initial crystal structure inside the model was disrupted and reorganized to form a new crystal structure. However, the migration time of the atoms was reduced during rapid cooling, which resulted in the inhibition of the grain growth.

**Figure 4:**
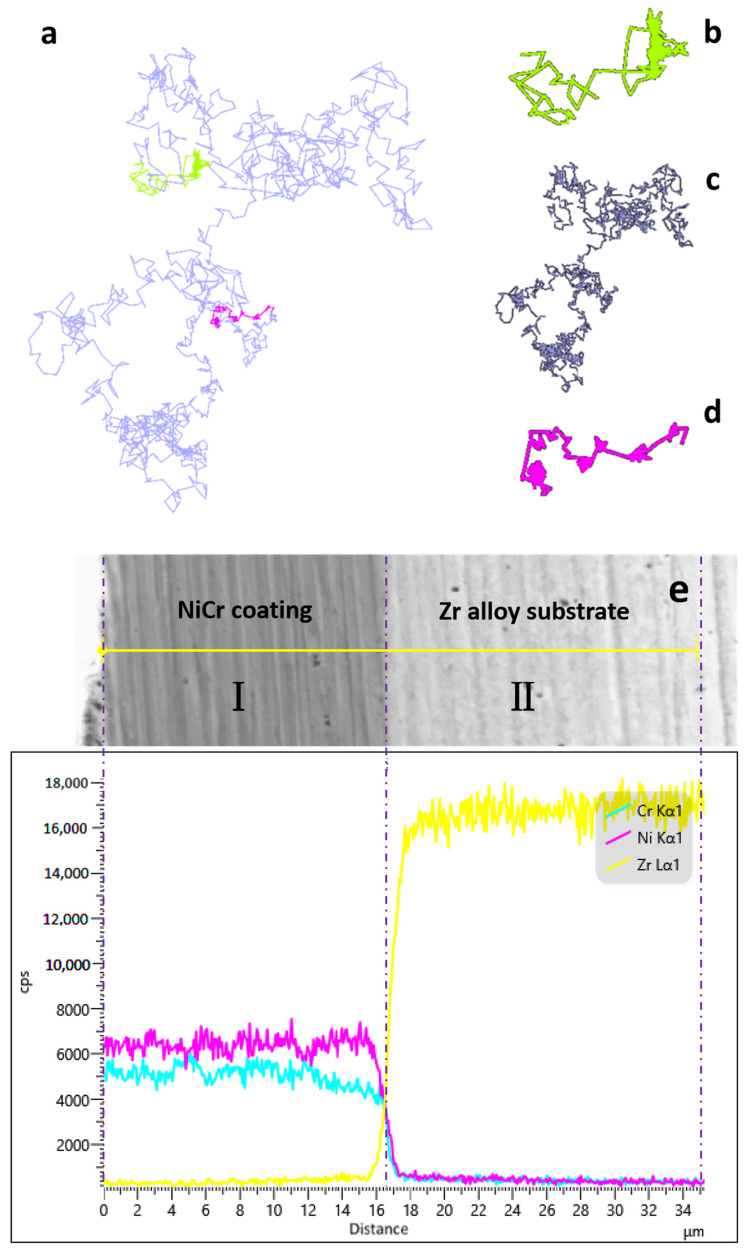

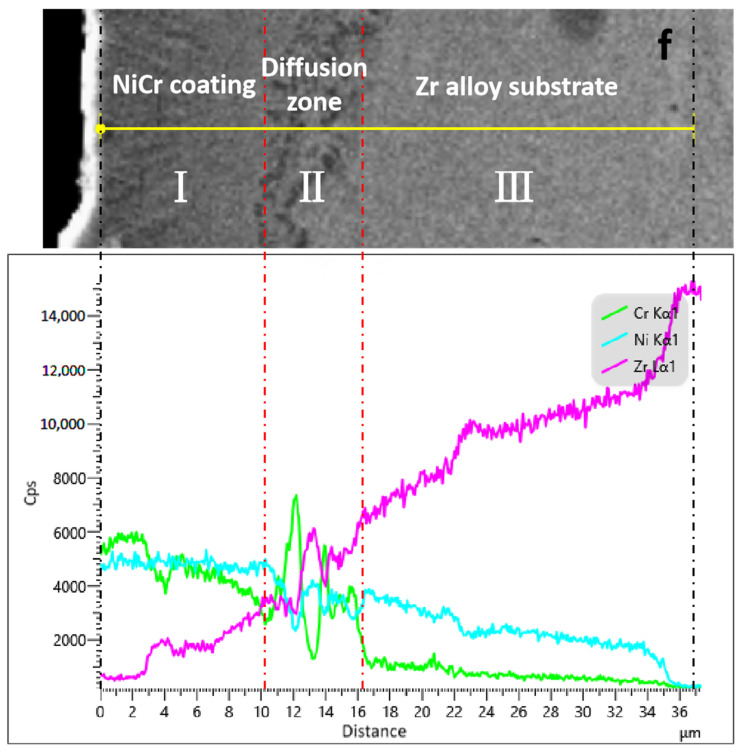
(**a**) Calibrated atomic motion trajectories; (**b**–**d**) the partial atomic trajectories of (**a**); (**e**) the EDS analysis of the cross-section of the NiCr alloy coating before LMM treatment; (**f**) the EDS analysis of the cross-section of the NiCr alloy coating after LMM treatment.

During the LMM process, the surface of the coating and substrate were remelted. The atoms ran with each other following the law of diffusion and were fused after solidification. The diffusion of elements was revealed by simulating the movement of atoms. Figure 4a demonstrates the trajectories of the calibrated atoms throughout the model operation. Figure 4b illustrates the motion trajectory of an atom detached from its original ordered position and changed to a disordered atom after heating. The initial thermal motion of the atoms was affected by the crystal structure. The atoms only migrated within a small range; hence, the initial part of the trajectory was dense. As the temperature continued to increase, the crystal structure was disrupted, the thermal motion of the atoms increased, and the migration of the atoms increased and became unstable [33]. Figure 4c shows the motion trajectories of the atoms during the disordered process when all the atoms were in a disordered state without lattice constraints and had a large range of motion. Figure 4d shows the trajectory of the atoms after the model started to crystallize. When the temperature decreased, the model started to form a crystal structure. As the model atoms were subjected to lattice confinement, both the migration and range of motion decreased, and the system eventually stabilized. As shown in Figure 4e, the EDS line scan of the specimen cross-section before LMM revealed a clear boundary between the coating and substrate portions. There was a sharp drop in the distribution of the elements in the figure, indicating that the different coating elements did not diffuse between each other. This corresponded to the characteristics of the coatings prepared using PVD technology. Figure 4f demonstrates the EDS line scan of the specimen cross-section after LMM treatment, where I is the NiCr alloy coating region, II is the metallurgically bonded region, and III is the Zr alloy substrate region. The atoms absorbed external energy and underwent migration and diffusion. The crystal lattice in the coating was destroyed and recombined to form a new crystal structure. The elements diffused into each other and solidified, transforming the physical bond between the coating and substrate into a metallurgical bond. The formation of metallurgical bonding zones enabled the coating to maintain excellent adhesion and enhanced the coating protection in high-radiation thermal shock environments [34].

In solid-state systems, the centrosymmetry parameter (CSP) is useful for measuring the local lattice disorder around an atom and can be used to characterize atoms as part of a perfect lattice or as local defects (dislocations, etc.). The CSP is defined as two adjacent vectors from the central atom to a pair of neighboring atoms. Its centrosymmetric parameters are defined as follows.

(4) $$\Delta =\frac{1}{{6d}_{0}^{2}}\sum {\left|R_{i}+R_{i+6}\right|}^{2},$$

where R_i_ is the displacement vector of the connections between an atom and its immediate neighbors. Among the relative neighboring vectors, there were six pairs for the FCC and four pairs for the BCC. Therefore, when setting the standard parameters, the FCC lattice was 12 and the BCC lattice was 8. The lattice in an ideal crystal is one, in which the vectors of the neighboring pairs cancel each other out and the CSP value is zero. On the contrary, the atomic sites in defective crystals usually have perturbed and non-centrally symmetric neighborhoods, under which the vectors are unsymmetrical and do not cancel each other out, and the CSP value is positive. By adjusting the appropriate threshold, small perturbations due to thermal displacement and elastic deformations can be permitted, which in turn filters out some of the atoms in the crystal defects.

**Figure 5:**
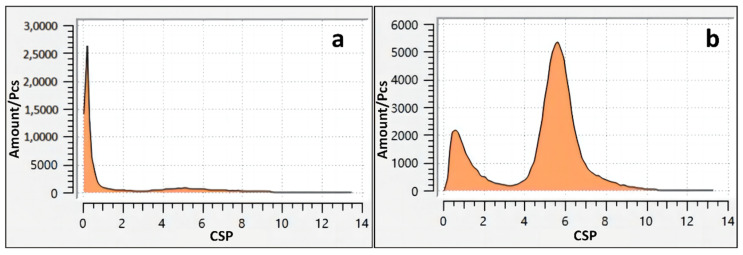
The centrosymmetric parameter map; (**a**) the initial state, (**b**) the final state.

**Figure 6:**
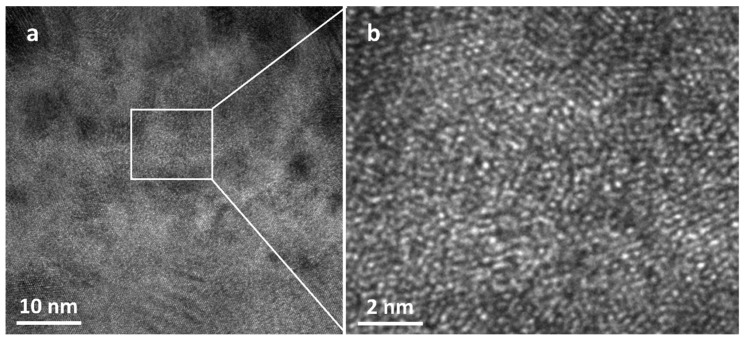
The TEM image of the coated specimen after LMM treatment; (**b**) is the partial enlargement of (**a**).

Figure 5a indicates the CSP of the initial state. The initial atoms were localized along the standard state with no delocalized particles, and the presence of the near dislocation lines and grain boundaries caused the CSP value to be mostly close to zero. As shown in Figure 5b, the existence of a large number of off-site particles, dislocation lines, and other defects in the model of the final state, led to the fact that the vectors of the atoms in the neighboring pairs in the model did not cancel each other. Therefore, the CSP value was positive and the number of disordered atoms increased. Figure 6 shows the TEM image of the specimen after LMM treatment. It was evident that when the CSP value was positive, there was an amorphous structure in the region, i.e., the relativity of the neighboring parameters around the atoms was destroyed, and the atoms were in a disordered state or were partially short-range ordered and long-range disordered.

Dislocations are a type of line defect resulting from the localized irregular arrangement of atoms in a crystal. There are various models for dislocations, including blade-type, screw-type, and hybrid dislocations. In this study, the dislocation line was perpendicular to the lattice slip direction in the blade-type dislocation. The atoms in the vicinity of the dislocation line in the screw-type dislocation were arranged in a helical shape, while in a hybrid dislocation, the dislocation line was not parallel or perpendicular to the lattice slip, and the angle between them was arbitrary. A dislocation line is the boundary line of an already slippery region in a crystal or lattice and can be of any shape. Lattice distortion occurred in the region near the dislocation line, and different lattice distortions were determined by the dislocation type in the lattice. To study the nature of dislocations, the Burgers vector b was introduced. This revealed the nature of the dislocations. Since s dislocation can have only one Burgers vector, the type of dislocation could be determined from the relationship between the Burgers vector and the dislocation line. The Burgers vectors of the edge dislocations were perpendicular to the dislocation line, those of the screw dislocations were parallel to the dislocation line, and those of the mixed dislocations were at a random angle to the dislocation line and had no distinctive features.

**Figure 7:**
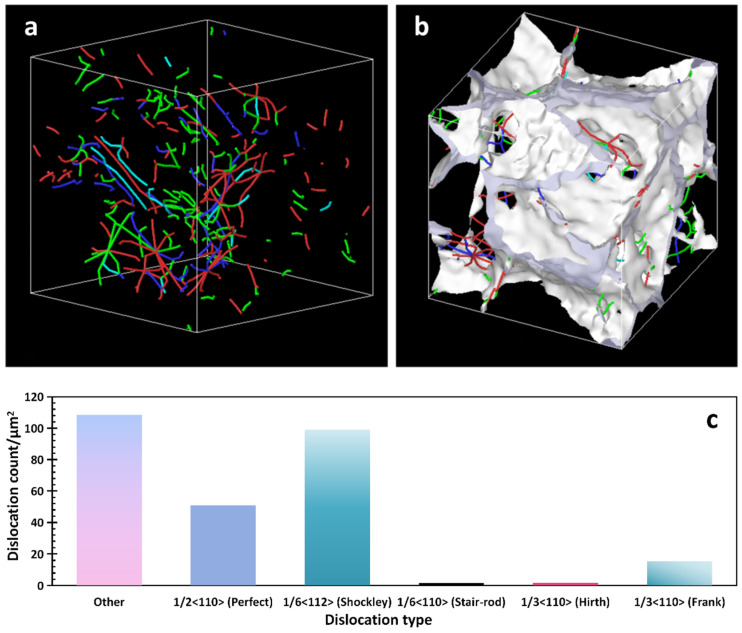
(**a**,**b**) diagrams of the dislocation distribution before the model operation; (**c**) diagram of the dislocation types before the model operation.

The dislocations in the model system were mainly FCC perfect and imperfect dislocations. The remainder of the dislocation types were set to “other”. Figure 7a demonstrates the initial distribution of the dislocation lines. Combined with the findings illustrated in Figure 7c, it can be determined that the dislocation lines of the BCC structure were mainly imperfect dislocations of 1/6 <112> and “other” type dislocations. The dislocation lines in the model contained edges, screws, and mixed dislocations.

**Figure 8:**
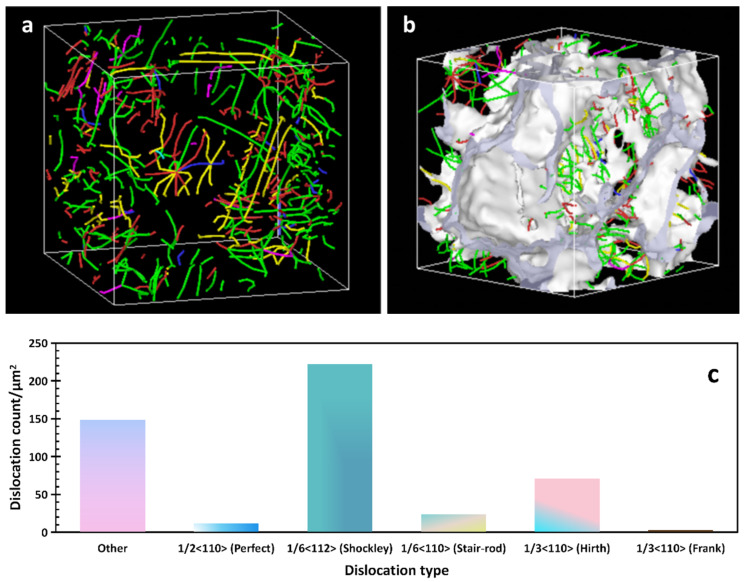
(**a**,**b**) diagrams of the dislocation distribution after the model operation; (**c**) diagram of the dislocation types after the model operation.

Figure 8 shows the dislocation information that was obtained when the model was at room temperature after undergoing the LMM process. The number of perfect dislocations in the FCC structures decreased, while the number of imperfect dislocations in 1/6 <112> increased by approximately 100% and the number of “other” dislocations increased by approximately 50%. As shown in Figure 8a, the number of dislocations increased after LMM treatment, and most of the dislocation lines accumulated near the grain boundaries. 

As illustrated in Figure 7b and Figure 8b, most of the dislocation lines were distributed in the region of the grain boundaries. Under the action of shear stress, many of the dislocations moved along the slip surface, and when encountering obstacles such as grain boundaries, fixed dislocations, and impurity particles, the dislocations were blocked, and a plugging group of dislocations was formed. If an external force was applied, the dislocation source generated dislocations once again, and the strength of the material increased when the dislocation motion was hindered [35].

The dislocation density is the total number of dislocation lines per unit area and is calculated by the following formula.

(5) $$\rho =\frac{n}{A\;\;},$$

where n is the total number of dislocation lines and A is the area where the dislocation lines are located. The common unit for the dislocation density is 1/m^2^.

**Figure 9:**
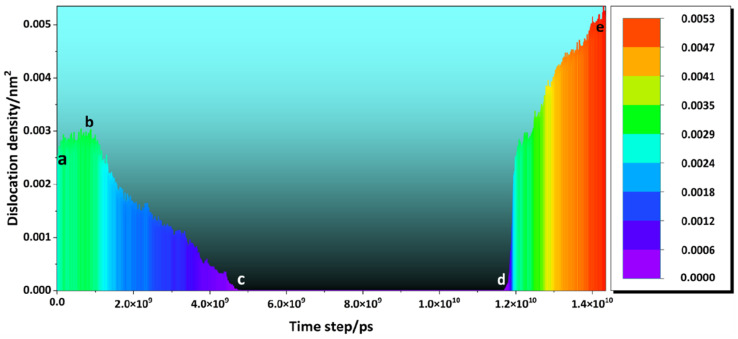
Dislocation density curve during the simulation.

As shown in Figure 9, the initial dislocation density of the model was 2.34 × 10^−3^ nm^−2^. In the preheating stage (sections a,b), the dislocation density increased slowly with the temperature. During the LMM process (sections b,c), the temperature increased dramatically and the atoms in the system absorbed energy and delocalized. The crystal structure was destroyed, and the dislocation density began to decrease. After melting the sample (sections c,d), the atoms in the system were transformed into a disordered state, and the dislocation density was zero. After the LMM process, the external energy and temperature decreased. The system began to supercool internally and nucleate, with the disordered atoms transforming into ordered atoms and forming a crystalline structure. With increasing strain, the grains nucleated continuously at the twin grain boundaries and gradually grew into the surrounding matrix, which prevented the migration of the grain boundaries and increased the dislocation density [36]. The final dislocation density of the system was 4.86 × 10^−3^ nm^−2^, which was an increase of 107.7% compared to the initial dislocation density.

**Figure 10:**
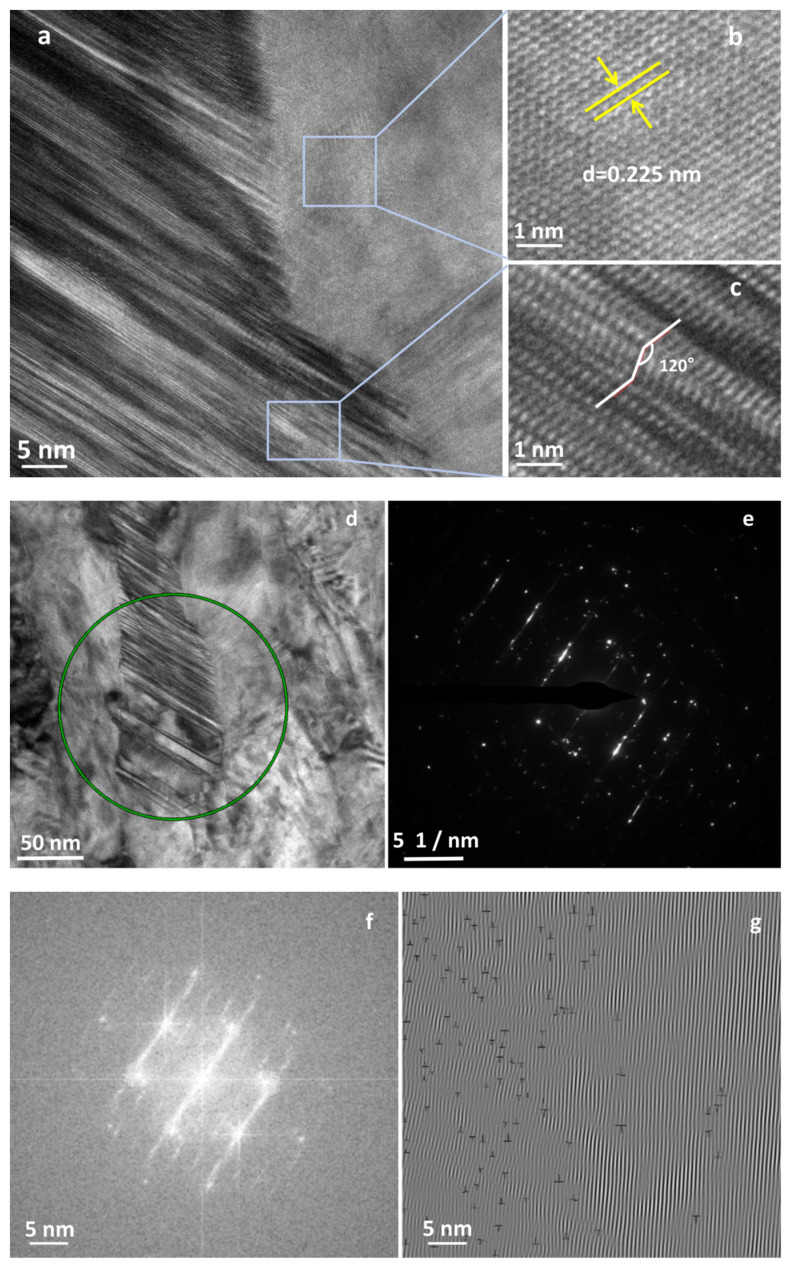
TEM diagrams of the sample after undergoing the LMM process: (**b**,**c**) show partially enlarged views of (**a**); (**e**) demonstrates the diffraction pattern of the green region in (**d**); (**f**) shows the Fourier transform of (**a**); and (**g**) indicates the inverse Fourier transform of (**a**).

Figure 10 shows a TEM image of the sample after LMM treatment. Obvious lattice fringes were present (Figure 10b), the atoms were arranged periodically according to a certain rule, and the lattice spacing was 0.225 nm. The twin structures formed during crystallization are illustrated in Figure 10c. The plastic deformation of the metallic materials was based on the nucleation and movement of the dislocations. Dislocation plugging is a common microscopic behavior in the plastic deformation of metals. The strain hardening of materials was mainly caused by the dislocation proliferation and the mutual obstruction of the dislocations. When the dislocations were subjected to obstacles, such as grain boundaries during the motion proliferation, they were plugged in front of the obstacles, forming dislocation plug clusters [37]. The twinning surface impeded the movement of the dislocations and the dislocation accumulation at the twinning boundary. When the dislocations entered or crossed a twinning surface, more external stress was required to break this surface [38]. The presence of stacking faults and twin structures in the sample is illustrated in Figure 10d, and the density of the dislocations in this region was considerably larger than that in the other regions. The twin grain boundaries impeded the dislocation motion, resulting in higher dislocation densities in their vicinity. The dislocations were blocked near the grain boundaries, and the density of the dislocations increased, at which time the hardness of the material increased with an increase in the dislocation density. Many of the dislocations interacted with each other and prevented further movement of the dislocations, thus increasing the strength of the material [39,40,41,42]. When the dislocation pileup reached a certain degree, the stress concentration increased, and the dislocations broke through the grain boundaries and barriers, resulting in plastic deformation or cracks. The calibrated dislocation density was 3.29 × 10^−2^ nm^−2^, as shown in Figure 10g. From the TEM image, it can be concluded that the distribution of the dislocations in the coating was not homogeneous, which led to an order of magnitude higher detection than the simulated results. During the LMM process, the dislocations were unevenly distributed along the temperature gradient and in the lap direction. Meanwhile, the dislocations interacted with twins and nanoparticles to exacerbate this phenomenon [43]. 

Moreover, the microhardness of the samples was analyzed at 10 randomly selected points on the surface of the samples. The results (in Table 4) demonstrated that the microhardness of the samples after LMM treatment was 475.9 HV. The hardness values were processed using the root mean square error (RMSE). The RMSE was obtained as 27.41, and a comparison of its average hardness value revealed that there was a large difference in the microhardness between the different regions of the coating surface after the LMM process. After LMM treatment, the porosity of the coating decreased, and the grain refinement dramatically improved the microhardness of the coating surface. In addition, the disordered interface inhibited dislocation nucleation and the dislocation motion of the material, thereby maintaining a high dislocation density inside the material. The uniform distribution of the dislocations suppressed the localized strains in the material, which in turn enhanced its strength [44,45].

**Figure 11:**
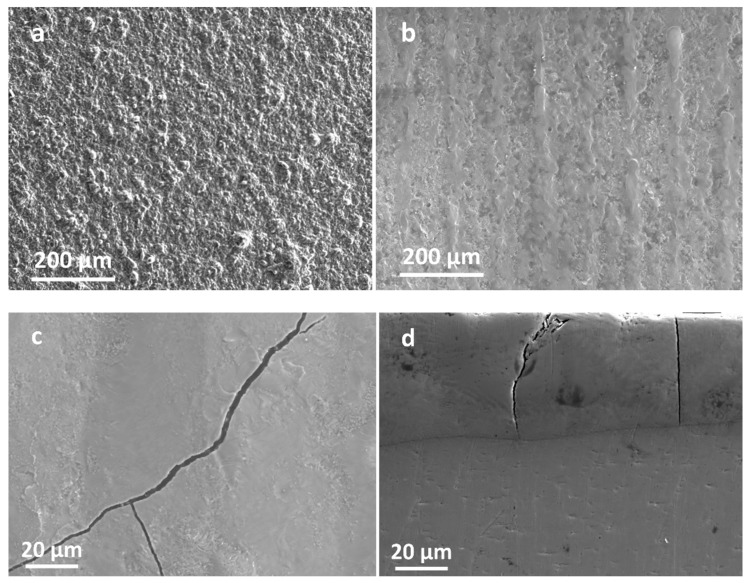
SEM morphology of the coatings: (**a**,**b**) surface morphology of the PVD coating and PVD–LMM coating, respectively; (**c**,**d**) cracks morphology of the coating surface and cross-section, respectively.

Figure 11 demonstrates the SEM morphology of the coating surface. Figure 11a displays the PVD coating, which had a generally flat surface. However, there were particles and droplets of various sizes on the surface of the PVD coating, and there were holes and gaps among the particles. During the preparation of the coating using PVD technology, the target material was heated using the cathode arc spot, which led to the formation of a molten pool on the target surface. As a result, the metal was vaporized due to the heat, and the molten droplets on the target material were bombarded by high-energy particles. These droplets were then transformed into particles, which splashed away from the target surface and were deposited onto the surface of the substrate. Figure 11b illustrates the PVD–LMM coating, and a conclusion can be drawn that the surface of the coating appeared flatter after LMM treatment. Remelting and condensing after LMM processing eliminated defects, such as large particles and liquid droplets on the surface of the PVD coating, preliminarily verifying the feasibility of the micrometer-sized protective coatings preparation. The coating remained undistorted under the impact of the laser beam. However, a small number of microcracks were present in some areas and a portion of the crack extension down to the substrate, as illustrated in Figure 11c,d. The large accumulation of dislocations at the twins caused the build-up of stress, which resulted in the formation of crack sources. Furthermore, the stress was not fully released during the rapid condensation process of the molten pool, which led to the further expansion of the cracks.

**Figure 12:**
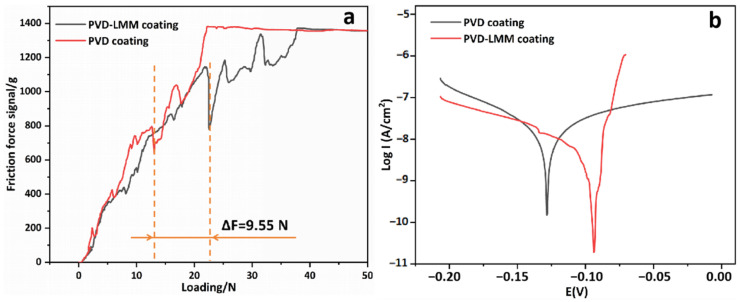
(**a**) Scratch test friction force signal diagram; (**b**) polarization curves.

The coating bonding properties were tested, and the results are shown in Figure 12a. The PVD coating failed under an applied pressure of 13.05 N, while the LMM treatment increased the coating bond force to 22.6 N and the film–base adhesion by 73.18%. After the LMM treatment, the coating remelted with the surface layer of the substrate, and the elements diffused into each other and fused upon condensation (Figure 4f). The coating changed from an initial adsorptive physical bond into a metallurgical bond. The augmented bonding strength of the film–base facilitated a better adhesion of the coating in the face of impact. The corrosion resistance of the coating before and after LMM treatment was tested and the polarization curves are shown in Figure 12b. The corrosion resistance of the coating significantly improved after LMM treatment. Defects such as the porosity and large droplets present in PVD coatings were eliminated by the LMM treatment. The disappearance of droplet defects was conducive to improving the flatness of the coating and reducing the contact area of the coating during corrosion. Additionally, it improved the densification of the coating by removing the pores. This directly eliminated the channel that corrosive liquids could enter, leading to an improvement in the corrosion resistance of the coating.

## 4. Conclusions

We applied PVD and LMM composite technologies to prepare NiCr alloy coatings on the surface of Zr alloys, which overcame the issue of low bonding among the coatings prepared using PVD technology and the base material. The molecular dynamics simulations of the LMM process for the NiCr coatings demonstrated that the dislocation lines were mainly stacked at the grain boundaries, and 1/6 <112> imperfect dislocations were dominant as the dislocation density increased by 107.7%. The TEM results showed that the dislocations in the twinning region were affected by the twin boundaries and slip surfaces that were plugged into their vicinity, and the density of the dislocations was considerably larger than that in the other regions. After LMM treatment, the mechanical properties and corrosion resistance of the coating were significantly improved. This PVD–LMM method shows promise for improving Zr alloys for practical applications under extremely harsh conditions over long-term periods. In the next work, we will address the phenomenon of the accumulation of high stresses due to the high energy input during the LMM process.

## Figures and Tables

**Table 1:** Target impurity content.

Element	Co	Si	Mg	Cu	Ti	Fe	Overall Amount
Content. (ppm)	90	48	45	52	32	220	≤0.1

**Table 2:** PVD process parameters.

Substrate Bias Voltage	Target Current	Deposition Time	Ar Gas Flow Rate	Duty Cycle	Target Base Distance	Substrate Temperature
−200 V	65 A	90 min	110 mL/min	50%	160 mm	300 °C

**Table 3:** LMM process parameters.

Laser Power	Scanning Speed	Defocusing Amount	Offset	Ar Gas Flow Rate
30 W	1500 mm/min	10 mm	0.045 mm	10 L/min

**Table 4:** Surface microhardness of the different samples.

Sample	Hardness Value/HV										Average Value/HV	RMSE
PVD–LMM	504.9	454.1	462.1	509.7	507.5	441.0	483.6	431.2	466.3	498.2	475.9	27.41

## Data Availability

Data are contained within the article.
